# Editorial: Advances in Soft Robotics Based on Outputs From IROS 2018

**DOI:** 10.3389/frobt.2020.00124

**Published:** 2020-08-26

**Authors:** Concepción A. Monje, Cecilia Laschi

**Affiliations:** ^1^Systems Engineering and Automation, Universidad Carlos III de Madrid, Leganés, Spain; ^2^Sant'Anna School of Advanced Studies, Pisa, Italy

**Keywords:** soft robotics, robust control, smart materials, morphological computation, modeling of soft robots

This Research Topic is devoted to publishing extended versions of the papers presented under the “Soft Robotics” keyword at the 2018 IEEE/RSJ International Conference of Intelligent Robots and Systems (IROS 2018) held on 1-5 October 2018 in Madrid, Spain.

IROS is a flagship conference in the field of Robotics. With more than 2,700 submissions and more than 1,250 accepted papers, the 2018 edition broke all records of previous Robotics congresses worldwide. With activities such as workshops, tutorials and special sessions, and late-breaking results, the conference brought a total number of 3,010 submissions.

Nowadays many research labs are focusing their activities on creating new robotic structures that are soft, not rigid, combining soft materials science and robotics to create new types of robots. Soft robots may exhibit abilities that were not possible before (Laschi et al., 2016) and that enhance robot applications in human-robot interaction, adaptation, and autonomous operation. Soft robotics can serve as a better alternative to rigid robots in natural environments and in human operations where safety and adaptability to uncertainties are fundamental requirements. Soft robots can adapt to variable environments and they can move adapting themselves to the requirements of the task. They can manipulate unknown objects that vary in size and shape (Hughes et al., 2016) and their soft conditions allow them to access and operate in confined spaces, to adapt their shape and even to grow (Del Dottore et al., 2019) and self-heal (Bilodeau and Kramer-Bottiglio, 2017). Soft robotics poses interesting challenges for research in several aspects from manufacturing (Schmitt et al., 2018) and control (Mena et al.; Muñoz et al., 2020).

Soft Robotics is a young yet productive field, attracting a growing interest from scientists in robotics and other disciplines. It has been calculated that the number of papers with the “soft robotics” keyword had a step-wise increase of 66% in 2012, and this trend continued with increases of 17% in 2013, 30% in 2014, and 35% in 2016 (Bao et al., 2018). *Frontiers in Robotics and AI* devotes a section to soft robotics (https://www.frontiersin.org/journals/robotics-and-ai/sections/soft-robotics) and as many as 20 Research Topics have been published since 2016. This trend also occurs at the IEEE robotics flagship conferences, like IROS 2018. The number of papers in soft robotics has been steadily increasing, and with them so have the conference sessions on this topic. Given the technical and scientific challenges of soft robotics, the number of workshops has also been increasing, for discussing the cutting-edge issues in soft robotics, spanning from design and development to modeling and intelligent control, from fabrication technologies to applications, and spanning across topics. Competitions are a productive way to set a standard for a new technology like soft robotics and to demonstrate its potential. After the first RoboSoft challenge in 2016 (Calisti et al., 2016), soft robotics competitions are held regularly at the main IEEE robotics conferences.

Soft Robotics has gathered a solid and vibrant scientific community that enlarges the boundaries of robotics in fields such as material science, chemistry, and biology. At the same time, the boundaries of robot abilities and applications are enlarged to tasks and scenarios that were previously out of reach, in medicine, personal assistance, services, and industry.

The aim of this Research Topic is to provide an insight of selected achievements from the Soft Robotics community. The Guest Editors invited authors of outstanding works who contributed to IROS 2018 to share their knowledge on key relevant issues that span from the implementation of the concept of morphological computation to the modeling of smart materials used in soft robotics, like SMA, up to the design and development of a bioinspired flexible neck, with a focus on how soft robotics can reach the milli-scale.

## Author Contributions

CM has contributed to this editorial with her expertise on design and modeling and control of robots based on soft links acting as humanoid robots' extremities. CL has contributed to this editorial with her expertise on design and modeling and control of soft bioinspired robots. All authors contributed to the article and approved the submitted version.

## Conflict of Interest

The authors declare that the research was conducted in the absence of any commercial or financial relationships that could be construed as a potential conflict of interest.

## References

[B1] Bao G. Fang H. Chen L. Wan Y. Xu F. Yang Q.. (2018). Soft robotics: academic insights and perspectives through bibliometric analysis. Soft Robot. 5, 229–241. 10.1089/soro.2017.0135 29782219 PMC5995266

[B2] Bilodeau R. A. Kramer-Bottiglio R. (2017). Self-healing and damage resilience for soft robotics: a review. Front. Robot. AI 4:48 10.3389/frobt.2017.00048

[B3] Calisti M. Cianchetti M. Manti M. Corucci F. Laschi C (2016). Contest-driven soft-robotics boost: the robosoft grand challenge. Front. Robot. AI 3:55 10.3389/frobt.2016.00055

[B4] Del Dottore E. Mondini A. Sadeghi A. Mazzolai B. (2019). Characterization of the growing from the tip as robot locomotion strategy. Front. Robot. AI 6:45 10.3389/frobt.2019.00045 PMC7805678 33501061

[B5] Hughes J. Culha U. Giardina F. Guenther F. Rosendo A. Iida F. (2016). Soft manipulators and grippers: a review. Front. Robot. AI 3:69 10.3389/frobt.2016.00069

[B6] Laschi C. Mazzolai B. Cianchetti M (2016). Soft robotics: technologies and systems pushing the boundaries of robot abilities. Sci. Robot. 1:eaah3690 10.1126/scirobotics.aah3690 33157856

[B7] Muñoz J. Monje C. A. Nagua L. F, Balaguer, C. (2020). A graphical tuning method for fractional order controllers based on iso-slope phase curves. ISA Trans. 10.1016/j.isatra.2020.05.045. [Epub ahead of print]. 32493576

[B8] Schmitt F. Piccin O. Barbé L. Bayle B. (2018). Soft robots manufacturing: a review. Front. Robot. AI 5:84 10.3389/frobt.2018.00084 PMC7805834 33500963

